# Synthesis of Flower-Like Cobalt–Molybdenum Mixed-Oxide Microspheres for Deep Aerobic Oxidative Desulfurization of Fuel

**DOI:** 10.3390/molecules28135073

**Published:** 2023-06-28

**Authors:** Xinxiang Cao, Ruijian Tong, Jingyuan Wang, Lan Zhang, Yulan Wang, Yan Lou, Xiaomeng Wang

**Affiliations:** 1 Laboratory for Development & Application of Cold Plasma Technology, College of Chemistry and Chemical Engineering, Luoyang Normal University, Luoyang 471022, China; xiangzi827@163.com (X.C.); 18639070635@163.com (Y.W.); ly210644026@163.com (Y.L.); w3542932371@163.com (X.W.); 2 School of Agriculture and Bioengineering, Heze University, Heze 274015, China; 3 Petrochemical Research Institute, PetroChina Company Limited, Beijing 102206, China; 4 School of Environmental Engineering and Chemistry, Luoyang Institute of Science and Technology, Luoyang 471023, China

**Keywords:** aerobic oxidative desulfurization, thiophenic sulfides, fuel, cobalt–molybdenum mixed oxides, flower-like microspheres, synergistic effect

## Abstract

Flower-like cobalt–molybdenum mixed-oxide microspheres (CoMo-FMs) with hierarchical architecture were successfully synthesized via a hydrothermal process and subsequent calcination step. The characterization results show that CoMo-FMs were assembled from ultrathin mesoporous nanosheets with thicknesses of around 4.0 nm, providing the composite with a large pore volume and a massive surface area. The synthesized CoMo-FMs were employed as catalysts for the aerobic oxidative desulfurization (AODS) of fuel, and the reaction results show that the optimal catalyst (CoMo-FM-2) demonstrated an outstanding catalytic performance. Over CoMo-FM-2, various thiophenic sulfides could be effective removed at 80–110 °C under an atmospheric pressure, and a complete conversion of sulfides could be achieved in at least six consecutive cycles without a detectable change in chemical compositions. Further, the catalytic mechanism was explored by conducting systemic radical trapping and transformation experiments, and the excellent catalytic performance for CoMo-FMs should be mainly due to the synergistic effect of Mo and Co elements.

## 1. Introduction

The emission of sulfur oxides from the large-scale combustion of sulfur-containing fuels represents one of the main contributors of air pollution that cause severe environmental issues [1]. At the present time, hydrodesulfurization technology (HDS) is primarily applied in chemical industry to eliminate the environmental hazards; however, it requires harsh reaction conditions, incurring a substantial cost of investment and operation [2,3]. The content of organic sulfur in different fractions of crude oil shows an increasing trend with the rise in their boiling points; as a result, the sulfur in straight-run diesel oil can reach up to more than 10,000 ppm. Among the sulfides in straight-run diesel, the sulfur contained in dibenzothiophene (DBT) and its derivatives could be in excess of 1000 ppm in most cases. Due to the synergy of the electron effect and steric hindrance effect, these refractory sulfides are extremely difficult to remove with the traditional HDS [4,5]. Given that, it is necessary to develop supplement technologies that show such advantages as a low energy consumption and high conversion efficiency regarding the refractory sulfides [6,7,8].

Among the emerging alternative desulfurization technologies, oxidative desulfurization (ODS) has been considered to be the preferred one for the effective removal of refractory thiophenic compounds under mild reaction conditions [9,10,11,12]. According to the principle of ODS, refractory sulfides can be converted to sulfones and/or sulfoxides and then removed by means of solvent extraction [13,14,15]. In the previous studies, H_2_O_2_ was frequently taken as the oxidizing agent [16,17,18] but, obviously, its usage on a large scale in industry will inevitably face serious safety concerns. Recently, substituting dioxygen (O_2_) for H_2_O_2_ to achieve aerobic oxidative desulfurization (AODS) has become a research hotspot [19,20,21,22,23]. As the most environmentally friendly oxidant, however, O_2_ is relatively chemically inert due to its triplet ground state (^3^O_2_) [24,25,26]. As a result, for AODS reaction systems, irreversible sacrificial agents had to be used [27,28].

In the past ten years, great progress has been made in the catalyst preparation for AODS. A wide variety of catalytic materials have been reported, such as metal oxides [29,30,31,32], boron nitride (BN) [33,34,35], reduced graphene oxide (rGO) [36], deep eutectic solvents (DESs) [37], metal–organic frameworks (MOFs) [38], and polyoxometalates (POMs) [37,39,40]. However, from the point of view of performance, cost, and stability, those existing catalysts still cannot meet the requirements of industrial applications [41]. Developing more active, reliable, and low-cost AODS catalysts is highly desirable [41]. Further, AODS is a kind of heterogeneous catalytic reaction occurring in complex gas–liquid–solid systems. The structure and morphology of catalysts greatly affect the mass transfer process between phase interfaces [42]. Therefore, for the development of high-performance catalysts, it is necessary to make an adjustment to the shape and size of catalysts from the macro-scale and improve the connectivity of pores at the meso scale, thus improving the mass transfer efficiency between gas–solid and liquid–solid phases [43]. Notably, mixed-metal oxides (MMOs) have achieved rapid advances in the catalysis science [44,45,46]. MMOs are highly diverse with a designable structure and composition [47]. For MMOs, under the scientific design, hierarchical porous structures can be developed and desired electronic structures for O_2_ activation can be achieved so as to meet the requirements of multi-phase reactions of AODS.

Herein, we report the successful synthesis of flower-like Co-Mo-O microspheres (denoted as CoMo-FMs) via a hydrothermal process followed by a calcination step. Surface morphologic structures, electronic structures, and crystalline phases as well as chemical compositions of the resulting samples with different Co/Mo dosages were systematically investigated. Further, the catalytic performance of CoMo-FMs for the aerobic oxidative desulfurization (AODS) of fuel was tested under mild conditions with dioxygen, O_2_, in air as the oxidant. In addition, catalyst stability tests were performed by six cycles of experiments. Furthermore, a catalytic mechanism of CoMo bimetallic oxides catalyzing the reaction of AODS was put forward.

## 2. Results and Discussion

### 2.1. Synthesis and Characterization of CoMo-FMs

**Figure 1:**
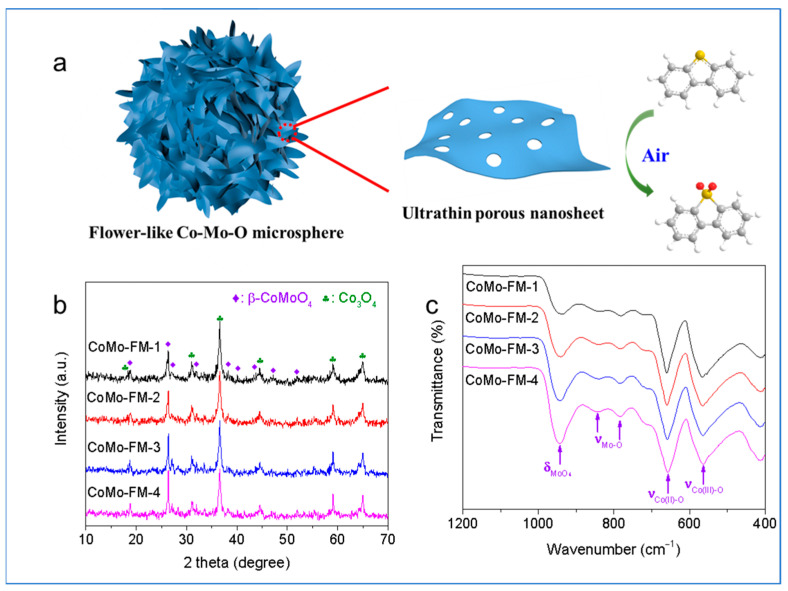
(**a**) Schematic illustration of AODS process over CoMo-FMs. (**b**) XRD patterns and (**c**) FT-IR spectra of CoMo-FMs.

CoMo-FMs were obtained by hydro-thermal synthesis with CoCl_2_·and (NH_4_)_6_Mo_7_O_24_ as precursors followed by calcination steps. While keeping the amount of CoCl_2_ constant, the effects of Mo/Co ratios on the chemical composition, morphologies, and catalytic performance of the resulting samples were systematically investigated (Figure 1a). We first conducted inductively coupled plasma–atomic emission spectroscopy (ICP-AES) to quantify the elemental contents within the samples (Table 1). As expected, the Mo/Co atomic ratio increased from 0.151 to 0.488 with an increase in its dosage ratio. The powder X-ray diffraction (XRD) spectra of CoMo-FMs (Figure 1b) mainly contain the diffraction peaks of Co_3_O_4_ and β-CoMoO_4_ [17,48]. In addition, the diffraction peak intensity of β-CoMoO_4_ increases with an increase in the Mo/Co ratio. Fourier-transform infrared (FT-IR) spectra collection was performed in the range of 400~1600 cm^−1^ to characterize the chemical structure of CoMo-FMs (Figure 1c). The characteristic peaks at 655 and 549 cm^−1^ are assigned to the stretching mode of Co(II)-O and Co(III)-O bonds in the oxide Co_3_O_4_ [49]. The peak present at 943 cm^−1^ should be attributed to the distortion of the MoO_4_^2−^ tetrahedron because of the distortion in CoMoO_4_. The peak at approximately 847 cm^−1^ is identified as a characteristic peak of Mo-O-Mo [50,51]. It can be observed that the intensity of peaks assigned to Mo species get enhanced with the increasing of the Mo/Co ratio, consistent with the XRD results.

**Figure 2:**
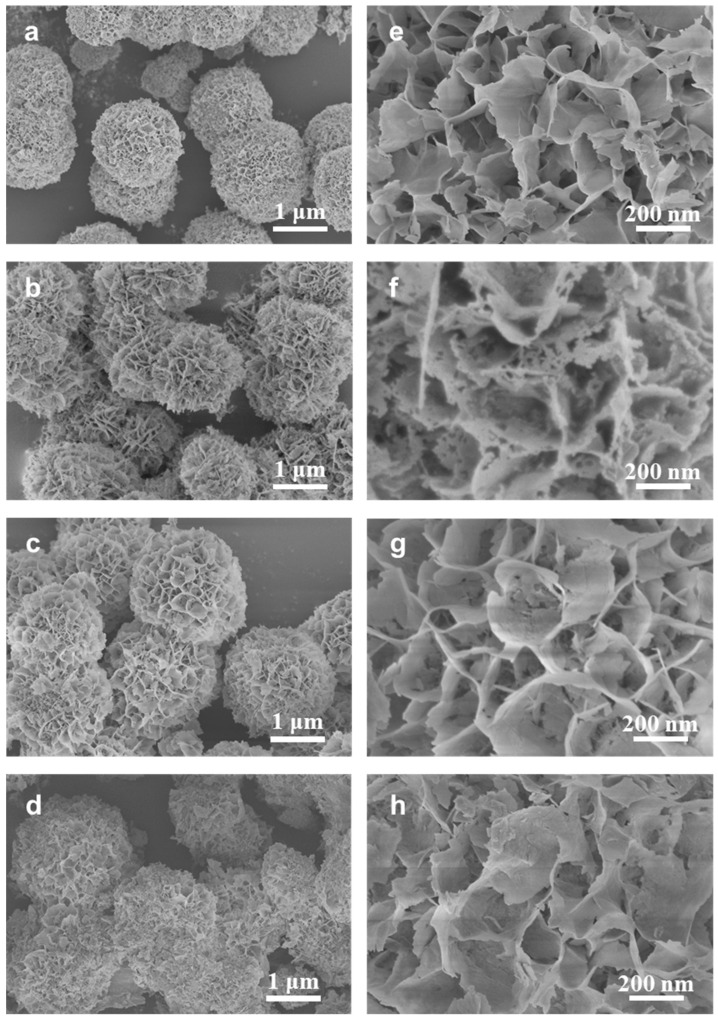
SEM characterization of the CoMo flower-like microsphere catalysts. (**a**,**e**) CoMo-FM-1, (**b**,**f**) CoMo-FM-2, (**c**,**g**) CoMo-FM-3, and (**d**,**h**) CoMo-FM-4.

To probe the morphologies of CoMo-FMs, scanning electron microscope (SEM) characterization was conducted. Figure 2a–d confirm that all the catalysts exhibit a flower-like microsphere structure. The particle size of CoMo-FM-1 and CoMo-FM-2 is approximately 1.5 μm, and it becomes larger for CoMo-FM-3. When further increasing the Mo/Co ratio, the materials do not form a regular microsphere. Figure 2e–h demonstrate that the micro-flowers are constructed by ultrathin nanosheets. It can be observed that the nanosheets in CoMo-FM-1 display a smooth surface but prove to be porous in CoMo-FM-2, whereas the nanosheets in CoMo-FM-3 and CoMo-FM-4 exhibit uneven thickness and a small amount of lumps.

**Figure 3:**
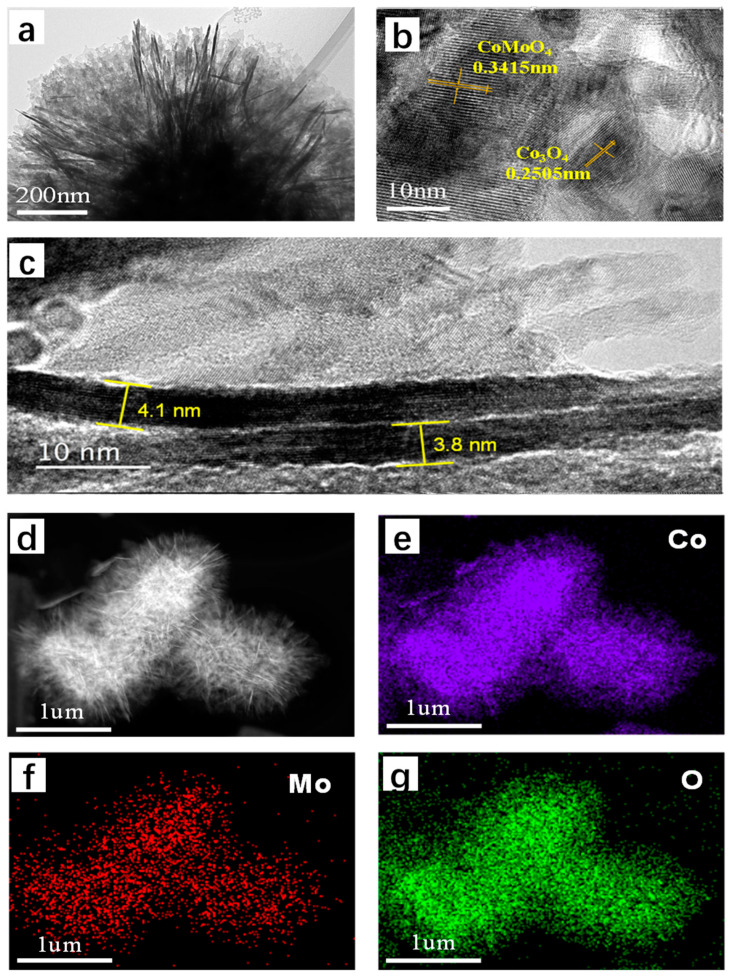
(**a**) TEM, (**b**,**c**) HRTEM, and (**d**) high-angle annular dark-field scanning TEM (HAADF-STEM) images of CoMo-FM-2 and corresponding EDS elemental mappings of (**e**) Co, (**f**) Mo, and (**g**) O.

**Figure 4:**
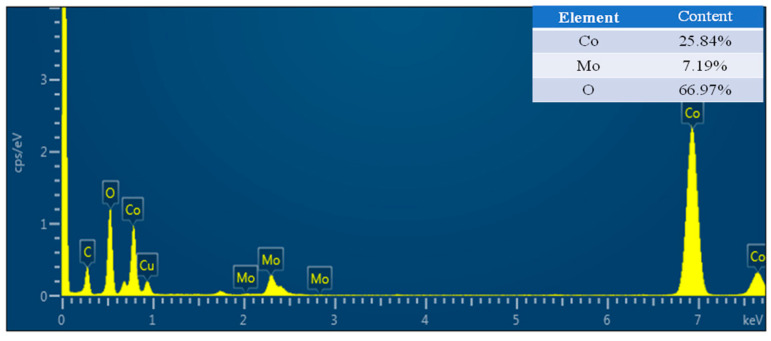
EDS results of CoMo-FM-2 performed by TEM.

The micro/nano-structure of CoMo-FM-2 was then observed by transmission electron microscopy (TEM). Figure 3a clearly demonstrates that the flower-like microsphere consists of nanosheets extending inside out. Furthermore, the nanosheets have an ultrathin thickness of approximately 4.0 nm (Figure 3c). As revealed by the high-resolution transmission electron microscopy (HRTEM) image in Figure 3b, two distinctive crystal forms with the lattice distance of 0.2505 nm and 0.3415 nm are observed, belonging to the (311) and (220) planes of Co_3_O_4_ and β-CoMoO_4_, respectively, conforming to the XRD results. Moreover, the HRTEM image of CoMo-FM-2 (Figure 3b) indicates the presence of mesoporous pores (highlighted by the red circles) on nanosheets. As shown in Figure 3d–g, the energy-dispersive spectroscopy (EDS) elemental mapping spectra of CoMo-MF-2 demonstrate that the elements, Co, Mo, and O uniformly distribute in the sample. It also can be inferred from the EDS results (Figure 4) that the contents of Co, O, and Mo account for 25.84%, 66.97%, and 7.19%, respectively, which are consistent with those determined by ICP-AES (Table 1).

**Figure 5:**
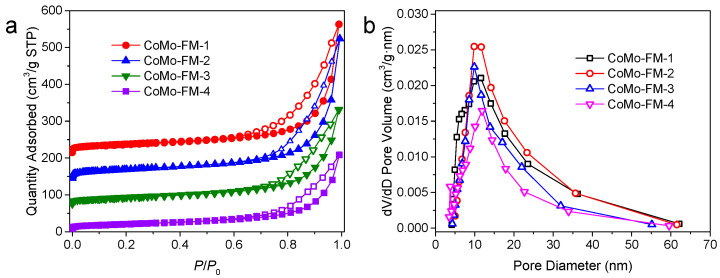
(**a**) N_2_ condensation adsorption/desorption isotherms and (**b**) the pore diameter distributions of CoMo-FMs.

A N_2_ physical adsorption–desorption test was conducted to investigate the pore structures of CoMo-FMs. Condensation adsorption/desorption isotherms of type V are designated for all four obtained samples (Figure 5a). Moreover, there is a hysteresis loop at relative pressure (P/P_0_) between 0.6 and 1.0 for each sample, indicating the existence of meso-pores or macro-pores. In addition, it can be seen in Figure 5b that all samples contain meso- and macro-pores with sizes distributed in the region of 5–60 nm. The mesopores in nanosheets and larger gaps between nanosheets are responsible for their hierarchical porous structure. It is noteworthy that CoMo-FM-2 possessed the largest pore size, pore volume, and surface area, covering up to 103.46 m^2^/g in all the samples (Table 2), which may significantly increase the number and accessibility of the active sites.

**Figure 6:**
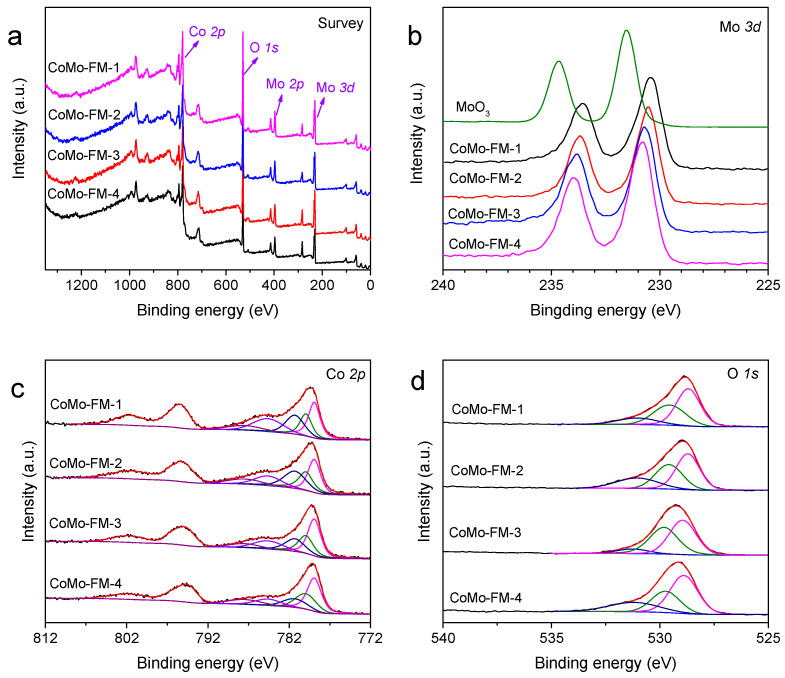
(**a**) XPS survey spectrums of CoMo-FMs and high-revolution XPS spectrums of (**b**) Mo3d, (**c**) Co2p, and (**d**) O_1s._

To investigate the surface chemical composition and chemical states of elements of CoMo-FMs, X-ray photoelectron spectroscopy (XPS) spectra were collected and are shown in Figure 6.

It can be discovered from the wide scan curves (Figure 6a) that CoMo-FMs contain three elements, which are Co, O, and Mo. As indicated by Figure 6b, doublet Mo 3d peaks are displayed at around 230.5 and 233.6 eV, assigned to levels of 3d_3/2_ and 3d_5/2_, which are different from the MoO_3_ reference (231.52 and 234.65 eV). The results demonstrate that Mo species in CoMo-FMs exist in a mixed-valence state of Mo(V) and Mo(VI), and Mo species gain electrons from Co species in the mixed-metal oxides. Consequently, with a decrease in the Mo/Co molar ratio, the Mo 3d peaks are observed to shift toward a lower binding energy. The high-revolution curves of Co 2p (Figure 6c) were smoothly fitted by setting the area ratio of 2p_1/2_ and 2p_3/2_ as 1:2. For the 2p_3/2_ component, the peaks at the point of 778.2 and 781.2 eV belong to Co(II) and Co(III) in Co_3_O_4_ [52], while the peak at 779.1 eV is assigned to the Co species in CoMoO_4_ [53]. It is also observed that the increase in the Mo/Co ratio reduced the proportion of Co(II), which further confirms the electrical effect of Co species in the oxides. Figure 6d presents the XPS spectra of O 1s, with three peaks at 531.4, 529.7, and 528.4 eV deconvoluted. The three peaks are assigned to adsorbed water, adsorbed oxygen, and lattice oxygen, respectively [54]. The proportions of adsorbed oxygen were then quantitively calculated and compared (Table 3). The CoMo-FM-2 and CoMo-FM-3 samples possessed a larger O_ad_/O_lat_ ratio, which indicates their higher concentration of oxygen vacancies.

### 2.2. Catalytic Performances of CoMo-FMs

**Figure 7:**
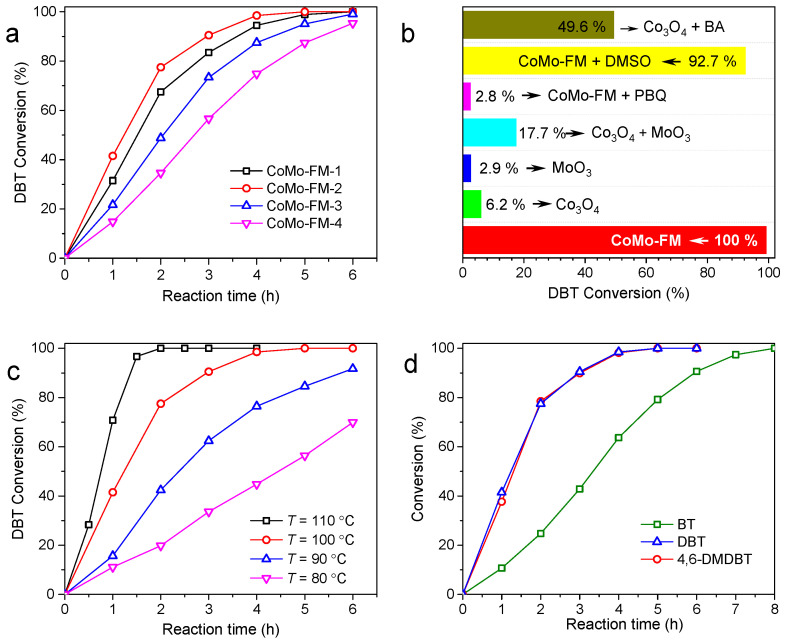
(**a**) Aerobic catalytic performance of CoMo-FMs. Reaction conditions: 20 mg catalyst, 20 g oil, 500 ppm DBT, and 100 °C. (**b**) Comparison of the effect of aerobic DBT oxidation in different catalytic systems. Reaction condition: 20 mg catalyst, 500 ppm DBT, 100 °C, and 4 h. (**c**) Catalytic performance of CoMo-FMs at different reaction temperatures in aerobic DBT oxidation. Reaction conditions: 20 mg catalyst, 20 g oil, and 500 ppm DBT (**d**) Exploring the catalytic effect of CoMo-FM-2 on different sulfides. Reaction condition: 20 mg catalyst, 20 g oil, 500 ppm initial sulfur content, and 100 °C.

Taking DBT as a model compound, the performances of CoMo-FMs for catalysis in deep aerobic oxidative desulfurization were evaluated. As shown in Figure 7a, all the catalysts display a noticeable sulfur removal effect. CoMo-FM-2 possesses the largest specific surface area and a relatively higher concentration of Mo(V) species and oxygen vacancies, performing the best in this work, achieving total transformation of DBT in 5 h. The hierarchical porous structure of CoMo-FM-2 is beneficial for reducing the mass transfer resistance of oxygen from the liquid–gas interface to the active sites of catalysts, while Mo(V) species and oxygen vacancies are supposed to be related to the catalytic behavior.

**Scheme 1:**
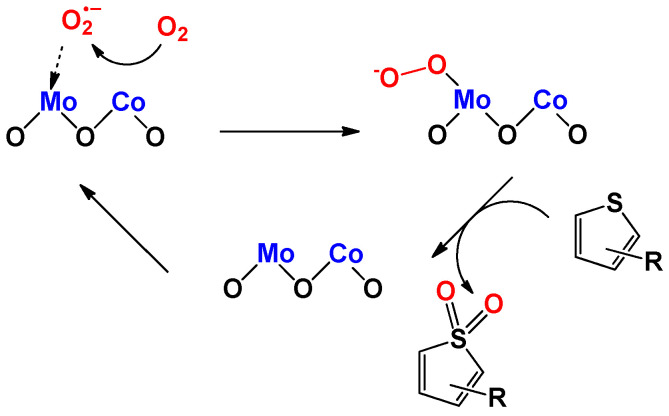
A proposed mechanism of thiophene oxidation over CoMo-FM.

The catalytic mechanism of CoMo bimetallic oxides in AODS was then explored. Co_3_O_4_ has been used as an active substance in the aerobic oxidation of alcohols. Nevertheless, sole Co_3_O_4_ as well as MoO_3_ manifested little activity upon the aerobic oxidation of DBT, as is indicated in Figure 7b. Control experiments were also carried out. Firstly, p-benzoquinone (PB) and dimethyl sulfoxide (DMSO) were charged into and employed as a radical scavenger of O_2_^•−^ and OH^•^, respectively, to identify the catalytic active substance [55,56]. The catalytic performance of CoMo-FM-2 deteriorated critically with the adding of PB, but was almost unaffected when DMSO was added. The results demonstrate that O_2_^•−^ instead of OH^•^ was an active intermediate substance during the catalytic reaction, whereas Co_3_O_4_ was also reported to be capable of activate O_2_ by forming O_2_^•−^. Then, Co_3_O_4_ and benzaldehyde (BA) were used as catalysts and showed obvious catalytic effect by achieving 49.6% of DBT conversion. In this reaction system, the generated O_2_^•−^ could be made further active by BA, forming active peroxyl species [27], and could then react with the thiophenic compounds. The above results reveal that both neat Co_3_O_4_ and CoMo bimetallic oxides can form active species with O_2_. However, the generated O_2_^•−^ is rich in electrons, and its direct reaction with thiophenic compounds is thus prohibited, while the Mo(V) species with oxygen vacancy is ready to interact with O_2_^•−^ to generate electrophilic species and hence realize the successful aerobic oxidation of sulfides (Scheme 1). Pure MoO_3_ does not have enough oxygen vacancies to form active species with O_2_^•−^, which also results in a low catalytic activity of the mixture of Co_3_O_4_ and MoO_3_. As shown in Figure 7c, as the temperature of the reaction rises, the conversions of DBT are clearly enhanced. It can be observed that complete DBT conversion is realized within 4 h at 100 °C.

Moreover, compared with those previously reported catalysts for the aerobic oxidation of DBT (Table 4), CoMo-FM-2 is evidenced to achieve a better catalytic effect under a similar reaction condition. In addition, it is observed that white needle-like crystals gradually precipitate in the oil phase during the cooling of the reaction system.

**Figure 8:**
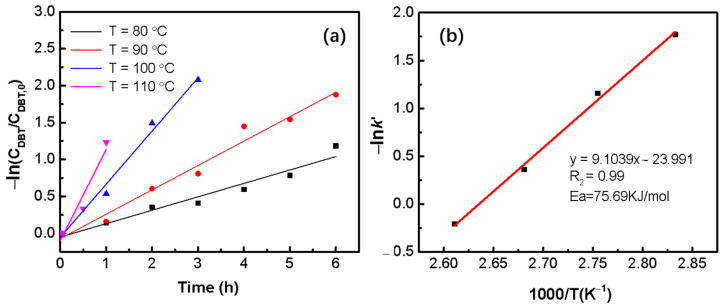
(**a**) Fitting of the kinetic data with pseudo-first-order model, and (**b**) the calculation of activation energy with Arrhenius equation for aerobic DBT oxidation over CoMo-FM-2.

We then collected the solid by filtration, dissolved them in ethanol, and analyzed the composition with GC-MS. It was evidenced that DBT was converted into dibenzothiophene sulfone (DBTO_2_). In addition, the first-order reaction kinetics were analyzed, and apparent reaction rate constants at different measurement temperatures were measured and then calculated (Figure 8a). Moreover, the apparent activation energy *E*a of aerobic DBT oxidation was found to be approximately 75.69 kJ/mol (Figure 8b).

**Figure 9:**
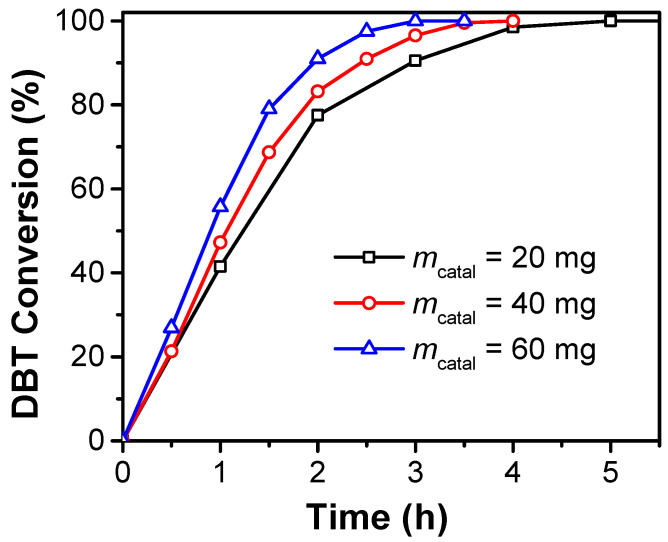
Aerobic catalytic performance of CoMo-FM-2 with different catalyst dosages for the oxidation of DBT. Reaction condition: initial DBT content = 500 ppm, *m*_oil_ = 20 g, T = 100 °C.

In addition, the effect of catalyst dosage on DBT conversion efficiency was also investigated. It was found that DBT conversion did not improve significantly with the increase in the amount of catalyst as revealed by Figure 9. Even when the catalyst dosage increased from 20 mg to 40 mg and then to 60 mg, the DBT conversion was only slightly improved. This is because the catalyst has a very low density, and excessive addition of the catalyst may lead to poor dispersion.

### 2.3. Catalytic Activities on Various Sulfides

As is known, the sulfur compounds in actual diesel oil are varied, among which, benzothiophene (BT), DBT, and their derivatives account for the most. Therefore, the performance of CoMo-FM-2 for the oxidation of other two sulfides, BT and 4,6-dimethyl dibenzothiophene (4,6-DMDBT), was also investigated in this study, and the results are shown in Figure 7d. It can be seen that the conversion of 4,6-DMDBT shows similarity with DBT, and complete conversions are reached within 5 h, while the performance of CoMo-FM-2 for BT removal was much lower. The difference in oxidative reactivity for the three thiophene compounds should be correlated with the electron density of sulfur atoms in their molecular structures [61]. In addition, gas chromatography–mass spectrometry (GC-MS) was employed to detect the products from the aerobic oxidation of DBT and 4,6-DMDBT, and the analysis results show that the two thiophene compounds were converted into corresponding sulfones.

### 2.4. Reusability of the Catalyst

**Figure 10:**
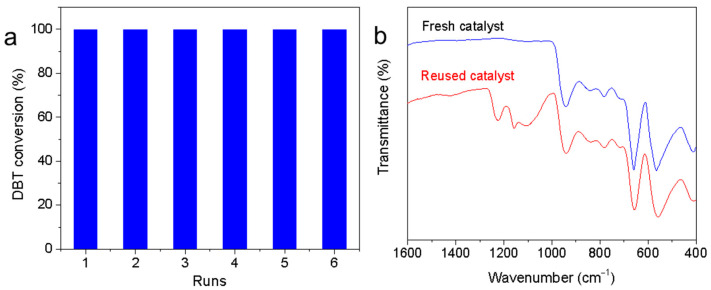
(**a**) Recycling performance of CoMo-FM-2 in aerobic oxidation of DBT (air flow rate of 20 mL/min) (**b**) FT-IR characterization of fresh and recycled CoMo-FM-2.

For a new catalyst, stability is a key performance index, and determines the reliability, economy, and ultimately the possibility of industrial applications of the catalyst. Given that, the catalyst CoMo-FM-2 was used to catalyze aerobic DBT oxidation for up to six cycles to test its stability. Briefly, CoMo-FM-2 was thoroughly mixed with model oil and stirring was maintained to catalyze the AODS reaction under the condition of 100 °C, 20 mL/min air, 20 mg catalyst, 20 g oil, and 500 ppm DBT for 4 h. After that, the slurry was centrifuged to collect the catalyst in the solid phase. Subsequently, the collected catalyst was washed by ethanol followed by vacuum drying for the next cycle. It is worth noting that the conversion of DBT remains 100% for each cycle experiment (Figure 10a), suggesting an excellent stability of the sample CoMo-FM-2. FTIR spectra were then used to compare the catalyst before and after recycle use and are shown in Figure 10b. After cycle usage for six times, CoMo-FM-2 still has clear FTIR characteristic absorption bands with no shift observed, while the new bands at 1250–1100 cm^−1^ are due to the generated sulfones adsorbed by the catalysts.

## 3. Materials and Methods

### 3.1. Chemicals

Benzothiophene (BT), dibenzothiophene (DBT), 4,6-dimethyl dibenzothiophene (4,6-DMDBT), cobalt (II) chloride hexahydrate (CoCl_2_·6H_2_O), ammonium heptamolybdate tetrahydrate ((NH_4_)6Mo_7_O_24_·4H_2_O), and hexamethylenetetramine (HMTA) were supplied by Aladdin Reagent Co., Ltd. (Shanghai, China). Ethanol (EtOH), benzaldehyde (BA), p-benzoquinone (PB), dimethyl sulfoxide (DMSO), and decahydronaphthalene were sourced from Tianjin Kermel Chemical Reagent Co., Ltd. (Tianjin, China). The purity of solid chemicals above was above 98%, the solvents were analytical grade, and all of them were used as received.

### 3.2. Preparation of CoMo-FMs

CoCl_2_·6H_2_O (1.5 mmol), (NH_4_)6Mo_7_O_24_·6H_2_O, and HMTA (10 mmol) were dissolved into 36.0 mL pure water and stirred to obtain a lavender solution, which was then homogenized and charged into a lining of a stainless-steel autoclave. After sealing, it was placed into an oven at 110 °C for 15 h followed by naturally cooling down to room temperature. The resulting slurry was centrifuged for collection of the solid product, which was then washed with pure water six times and followed by ethanol washing for one time. After drying at 60 °C for 8 h, the sample was subjected to calcination at 450 °C for 2 h to obtain CoMo bimetal oxides (denoted as CoMo-FM-x, x =1, 2, 3, and 4, with the dosage of (NH_4_)6Mo_7_O_24_·6H_2_O as 0.05, 0.10, 0.15, and 0.20 mmol, respectively).

### 3.3. Test of Catalytic Activity

Briefly, a sulfide (BT, DBT, or 4,6-DMDBT) was firstly dissolved in decahydronaphthalene to prepare a model oil with a sulfide concentration of 500 ppm. For the process of desulfurization experiments, the model oil (20 mL) and a certain amount of catalyst were placed into a 100 mL three-necked flask that was connected with a reflux condensing tube, a gas injector, and a port for the subsequent liquid sampling. Subsequently, the mixed solution was stirred continuously at 400 rpm at a desired temperature. Air was passed through a drying column to remove trace water prior to being bubbled into the liquid phase under atmospheric pressure for the reaction to be initiated. Sampling from the three-necked flask was performed at intervals for analyzing and determining the concentration of the residual sulfide by a GC-FID (HP-5 7820, Zhongke Huifen Instrument Co., Ltd., Beijing, China). For each AODS reaction, the experiment was repeated at least three times.

### 3.4. Characterization of Materials

The element content of Co and Mo in CoMo-FMs was quantitatively determined using the method of inductively coupled plasma–atomic emission spectroscopy (ICP-AES) (ICPE-9000, Shimadzu Co., Kyoto, Japan) Morphology of all the resulting catalysts was observed by a field emission scanning electron microscope (FE-SEM, XFlash 6100, Bruker, Karlsruhe, Germany). Powder X-ray diffraction (XRD) patterns of CoMo-FMs were identified by a D/max-2500V X-ray diffractometer (Rigaku Corp., Tokyo, Japan) using a Cu Kα (λ = 0.15418 nm) radiation source. Transmission electron microscopy (TEM), high-angle annular dark-field scanning TEM (HAADF-STEM), and energy-dispersive X-ray spectrometry (EDS) observations were conducted by using a transmission electron microscope (Talos F200X, Thermo Fisher Scientific, Waltham, MA, USA) equipped with an EDS equipment and a digitally acquired STEM imaging system. The Fourier-transform infrared (FT-IR) spectra of samples were obtained using a Nicolet is50 FT-IR spectrometer (Thermo Fisher Scientific, Waltham, MA, USA). The chemical states of several typical elements in CoMo-FMs were evaluated by X-ray photoelectron spectroscopy (XPS), performed on an X-ray photoelectron spectrometer (EscaLab Xi, Thermo Fisher Scientific, Madison, WI, USA). N_2_ physical adsorption/ desorption test was performed by a fully automated apparatus of physical adsorption (ASAP 2020 Plus HD88, Micromeritics Co., Norcross, GA, USA).

## 4. Conclusions

In summary, flower-like Co–Mo–O microspheres with hierarchical architecture were synthesized by a hydrothermal process followed by a calcination step. The micro spherical catalysts comprise Co_3_O_4_/CoMoO_4_ ultrathin porous nanosheets. It is shown that the diameter of the microspheres, the thickness of the nanosheets, and the diameter of channels on nanosheets are around 1.5 μm, 4 nm, and 10 nm, respectively, which makes the catalyst show an interconnected porous structure as well as a large pore size. The catalyst CoMo-FM-2 with a Mo/Co ratio of 0.265 shows the best catalytic performance for the AODS reaction; in particular, it could achieve the complete conversion of DBT and 4,6-DMDBT at 100 °C using atmospheric O_2_ as the oxidant source. It is worth noting that CoMo-FM-2 maintains almost constant catalytic activity for the AODS reaction during a six-cycle stability test. Radical trapping and transformation experiments reveal the synergistic effect of cobalt and molybdenum and the catalytic mechanism of the Co–Mo–O microspheres for the AODS reaction. Accordingly, the flower-like Co–Mo–O microspheres can be applied as robust and durable catalysts for AODS, and our results also reveal the significance of a rational design of a hierarchical structure for heterogeneous catalysis.

## Figures and Tables

**Table 1:** Elemental percentage of CoMo-FM determined by ICP-AES.

Sample	Elemental Percentage (atm.%)		Mo/Co Ratio
	Co	Mo	
CoMo-MF-1	29.22%	4.42%	0.151
CoMo-MF-2	25.93%	6.87%	0.265
CoMo-MF-3	22.88%	8.51%	0.372
CoMo-MF-4	21.36%	10.43%	0.488

**Table 2:** Porous texture of CoMo-MFs.

Sample	Specific Pore Volume /cm^3^ g^−1^	Specific Surface Area /m^2^ g^−1^	Mean Pore Size /nm
CoMo-FM-1	0.55	97.48	17.48
CoMo-FM-2	0.59	103.46	17.55
CoMo-FM-3	0.4	82.95	15.82
CoMo-FM-4	0.32	72.81	14.01

**Table 3:** Summarization of chemical states of Mo and O determined by XPS.

Sample	Mo 3d_5/2_	O_1s_		
		O_lat_	O_ad_	Water_ad_
CoMo-FM-1	230.44	528.68 (51.34%)	529.55 (34.59%)	531.01 (14.07%)
CoMo-FM-2	230.48	528.71 (44.54%)	529.57 (33.44%)	531.03 (22.02%)
CoMo-FM-3	230.61	528.89 (48.94%)	529.73 (43.67%)	531.19 (7.39%)
CoMo-FM-4	230.78	528.94 (50.43%)	529.79 (27.65%)	531.25 (21.92%)

**Table 4:** Comparation of catalytic performances with different catalysts.

Entry	Catalyst	Substrate	Oxidant	Reaction Conditions ^a^	Conversion	Ref.
1	CoMo-FM-3	DBT	Air	100 °C, 20 mg/20 g, 4 h	100%	This work
2	(NH_4_)_5_H_6_PV_8_Mo_4_O_40_	DBT	O_2_	100 °C, 20 mg, 6 h	88.4%	[57]
3	r-GO	DBT	O_2_	140 °C, 5 mg/25 mL, 4 h	100%	[34]
4	MoO_x_/MC-600	DBT	O_2_	120 °C, 10 mg, 8 h	100%	[38]
5	Ce–Mo–O	DBT	O_2_	100 °C, 100 mg, 6 h	100%	[29]
6	(Cu-Co)(salen)Y	DBT	O_2_	100 °C, 200 mg, 4 h	97.6%	[58]
7	MFM-300(V)	DBT	O_2_	120 °C, 0.75 g/L, 5 h	99.6%	[59]
8	Ch_x_Na_5-x_IMo_6_O_24_	DBT	O_2_	100 °C, 0.1 mmol/6 mL, 5 h	100%	[60]
9	MIL-101	DBT	O_2_	120 °C, 500 mg/L, 15 h	100%	[35]

^a^ Reaction conditions: reaction temperature, catalytic dosage/model oil volume, reaction time.

## Data Availability

Not applicable.
